# Robust Spatial–Spectral Squeeze–Excitation AdaBound Dense Network (SE-AB-Densenet) for Hyperspectral Image Classification

**DOI:** 10.3390/s22093229

**Published:** 2022-04-22

**Authors:** Kavitha Munishamaiaha, Gayathri Rajagopal, Dhilip Kumar Venkatesan, Muhammad Arif, Dragos Vicoveanu, Iuliana Chiuchisan, Diana Izdrui, Oana Geman

**Affiliations:** 1 Department of Electronics and Communication Engineering, Sri Venkateswara College of Engineering, Chennai 602117, India; kavithamunisamy@gmail.com; 2 Department of Computer Science Engineering, Vel Tech Rangarajan Dr. Sagunthala R&D Institute of Science and Technology, Chennai 600062, India; vdhilipkumar@veltech.edu.in; 3 Department of Computer Science and Information Technology, The University of Lahore, Lahore 54590, Pakistan; muhammad.arif@cs.uol.edu.pk; 4 Electrical Engineering and Computer Science Faculty, Stefan cel Mare University, 720229 Suceava, Romania; dragos.vicoveanu@usm.ro (D.V.); iuliana.chiuchisan@usm.ro (I.C.); diana.izdrui@usm.ro (D.I.)

**Keywords:** squeeze–excitation AdaBound dense network (SE-AB-DenseNet), hyperspectral image (HSI) classification (HSIC), cutout regularization

## Abstract

Increasing importance in the field of artificial intelligence has led to huge progress in remote sensing. Deep learning approaches have made tremendous progress in hyperspectral image (HSI) classification. However, the complexity in classifying the HSI data using a common convolutional neural network is still a challenge. Further, the network architecture becomes more complex when different spatial–spectral feature information is extracted. Usually, CNN has a large number of trainable parameters, which increases the computational complexity of HSI data. In this paper, an optimized squeeze–excitation AdaBound dense network (SE-AB-DenseNet) is designed to emphasize the significant spatial–spectral features of HSI data. The dense network is combined with the AdaBound and squeeze–excitation modules to give lower computation costs and better classification performance. The AdaBound optimizer gives the proposed model the ability to improve its stability and enhance its classification accuracy by approximately 2%. Additionally, the cutout regularization technique is used for HSI spatial–spectral classification to overcome the problem of overfitting. The experiments were carried out on two commonly used hyperspectral datasets (Indian Pines and Salinas). The experiment results on the datasets show a competitive classification accuracy when compared with state-of-the-art methods with limited training samples. From the SE-AB-DenseNet with the cutout model, the overall accuracies for the Indian Pines and Salinas datasets were observed to be 99.37 and 99.78, respectively.

## 1. Introduction

Hyperspectral imaging is the most popular monitoring tool of the Earth’s surface [1]. It consists of hundreds of spectral bands which are used to identify the physical and chemical properties of objects on Earth. With the advancement of hyperspectral sensor applications, it is easy to obtain images with a high level of spectral and spatial resolution information. HSI is widely used in a variety of applications, including mineralogy, agriculture, urban development, resource management, and the environment [2]. In the above-mentioned applications, classification is a fundamental step in assigning a particular class to each pixel, and this is the trending topic in the remote sensing community [3]. Support vector machines (SVMs) [4] with (72.84 overall accuracy), Bayesian models [5] with (69.35 overall accuracy), and k-nearest neighbor (KNN) [5] (90.05 overall accuracy) are examples of early machine learning techniques based on spectral information that were commonly employed in HSI classification. However, the HSI frequently contains redundancy or uneven noisy spectral bands due to the impact of the sensing devices and imaging mechanism [6]. Meanwhile, in the HSI area, the amount of training data is frequently limited in comparison to the number of spectral channels provided, making classifiers susceptible to overfitting [7]. However, the core complexity of HSI data and the insufficiency of labeled training samples usually challenge the efficiency of the classification in the HSI.

In the past, a large number of HSI classification methods have been proposed, mainly supervised machine learning methods, which have been utilized for HSI classification [8]. The advantage of using supervised classification is that it achieves enhanced classification accuracy with fewer training samples than unsupervised methods. Basically, there are two approaches to supervised classification methods: shallow classifiers and deep learning classifiers. As HSI provides an abundance of spectral and spatial information, it has become important to extract the salient features from them [8].

For HSI classification, several approaches have been presented; shallow classifiers work in two stages: the first is to extract features, and the second is to train them on the classifier [9]. K-nearest neighbor (KNN) and decision tree [8] are examples of supervised approaches. The difficulty of reducing the dimension becomes a major threat in classification [9] because multinomial logistic regression [8] typically uses high-dimension spectral information alone. To overcome this issue, many feature extraction methods were followed, such as principal component analysis (PCA) [10], independent component analysis (ICA) [11], and linear discriminant analysis (LDA) [12]. PCA is the most widely utilized of these approaches, which selects spectral bands following a modification based on the statistical variance. The study [4] employed randomized PCA to find principal components that contained 99% of the variance along a spectral dimension and achieved extremely significant accuracy with a deep CNN. The support vector machine (SVM) [4] method was significantly used for HSI classification with shallow architecture. SVM shows low sensitivity to the input data with large dimensions and a smaller sample size. A better performance measure is found in SVM when compared with other traditional classifiers. Moreover, there is redundancy in the spectral bands caused by sensors in the HSI [6]. Further, there is a limited number of training samples when compared with existing spectral bands. This leads to the classifier model being overfit [7]. The problem discussed makes the shallow approaches to provide an inefficient result throughout. If the amount of training data and accessible spectral channels are both minimal, the number of spectral characteristics (bands) grows and a significant number of training samples is necessary to classify. Spectral angle mapping (SAM) [13,14] is a method that compares the angle between the spectral directions of the correlation results and test pixels, hence no need for a huge number of training samples Thus, for hyperspectral imaging, the SAM approach is one of the best appealing classifications. However, in the SAM approach, there is a significant degree of error involved when the differences between the classes are captured by the variance of the input image as well as the differences between the orientations of the pixel spectra.

Recently, deep learning-based approaches [15,16] have the advantage of learning the parameters both automatically and sequentially. This has increased the research interest in solving the image classification problem. In order to invariantly discriminate the feature extraction process for various applications, including image classification, image segmentation, object detection, and natural language processing [17], deep learning has proven to be a boon in research. Deep learning approaches are alleged to have the capability of illustrating spatial–spectral features of HSIs in a prominent way and achieving higher classification accuracy than supervised shallow classifiers. The recent improvement of DL methods, deep belief networks [16], stacked auto-encoder (SAE) [18], convolutional neural networks (CNN) [1], and recurrent neural networks (RNN) [1] have been used in deep spectral classifiers for HSI. Among the deep learning models mentioned above, deep CNN-based approaches achieve better performance in terms of accuracy. It is challenging to establish a universal feature extraction approach employing such a method for complicated and varied hyperspectral data. A suitable method for feature extraction is a convolution neural network that can learn features from HSI on its own. CNN is a prominent model for carrying out a good rapport between spatial and spectral image classification.

In addition, the fully utilized spatial–spectral CNN-based classifier was first designed [19] with PCA, 3-D CNN, and logistic regression. The inputs were 3-D data and extracted spatial–spectral features for HSI classification. By automatically extracting features from hyperspectral images, HSI classification models based on 1D-CNN [20] or 2D-CNN [21] can obtain significant classification results, but at the cost of some spatial or spectral information loss. The 3D-CNN, which was previously used to handle video data, is introduced to HSI classification in order to fully exploit spatial and spectral information in hyperspectral images at the same time. The 3D-CNN has a higher computational overhead than 2D-CNN, but it can better learn spectral information inside a hyperspectral image, resulting in improved classification results. Hence, 3D-CNN has been widely used in HSI classification, and several improved models have been implemented based on it.

Yue et al. [19] developed a 3D-CNN model with 3D convolutional layers and 3D pooling layers that improved classification performance by delving into spatial–spectral features. Based on this, deep networks provide more robust features, and the entire network must be carefully designed to simulate a significant increase in the number of parameters. Pan et al. [22] constructed the FD-SSC (fast dense spectral–spatial convolution network) by introducing a dense block into SSRN and employing dense connections. With the use of a dense connection, FD-SSC improved feature propagation and reuse, allowing for the extraction of deeper hierarchical spatial–spectral characteristics. Structured innovation, in addition to the sensible use of varied residual connections, is an important part of the system fine tuning of CNN models for hyperspectral classification. Zhong et al. [23] took advantage of residual connections in spectral feature learning and created a deeper network (Res-2D-CNN) that allowed for the extraction of broader and more abstract features. Ahmad et al. [24] added residual blocks to 3D-CNN and created Res-3D-CNN to improve spatial–spectral feature learning. Zhu et al. [25] created SSRN (spectral–spatial residual network) from unstructured hyperspectral data without pre-processing and reducing the dimensionality. They separated the deep feature learning technique into discrete spatial feature learning and spectral feature learning and included residual connections across the network. SSRN learned more discriminative features, and the separated feature learning method will have a big impact on hyperspectral classification research in the future. Hyperspectral researchers have recently paid more attention to dense interconnections [26]. A dense connection minimizes network parameters by using a modest convolution kernel number and enables effective feature reuse by concatenating feature maps, both of which help to avoid model overfitting. HybridSN, a new hyperspectral spatial–spectral feature extraction pattern presented by Roy et al. [27], is based on the merging of 3D-CNN and 2D-CNN. HybridSN accepts hyperspectral data as input after dimensionality reduction and has a low computational overhead. It concatenates the feature maps generated in the spectral dimension by three successive 3D convolutional layers and then uses a 2D convolutional layer to improve spatial feature learning. HybridSN obtained extraordinarily high classification accuracy despite having only four convolutional layers, revealing the 3D-2D-CNN model’s enormous promise in hyperspectral classification. Yu et al. [26], who won the 2017 ImageNet Large Scale Visual Recognition Competition, created squeeze-and-excitation networks and incorporated the attention mechanism into the image classification network. Zhang et al. [20] developed a spatial–spectral squeeze-and-excitation (SSSE) module that automatically learns the weight of distinct spectral and neighboring pixels to emphasize the important features and suppress the unimportant ones, resulting in better classification accuracy. Wang et al. [28] introduced an attention module (the squeeze-and-excitation block) to emphasize effective features in spectral bands as the dense correlation module used for shallow and intermediate feature extraction, and then fed it to further deep feature extraction. In the HSI classification model, the attention mechanism is used to find more appropriate feature patterns in the spectral or spatial dimensions. However, there is no established theory for the specific application of the attention mechanism, such as the location and calculation methods, and more research is needed.

Fang et al. [29] presented a fully convolutional layer fusion network (FCLFN) to categorize HSIs by combining characteristics derived from all convolutional layers. Despite this, FCLFN uses a simple CNN model for feature extraction, which has issues with vanishing gradients and decreased accuracy when learning more discriminative features [30]. For HSI classification, in [30,31] presented a densely connected CNN (DenseNet), which divides the network into dense blocks and establishes short-cut interconnections between layers within each block. This connectivity arrangement eliminates the vanishing gradient problem and enables the HSI categorization of several characteristics from distinct layers. However, only layers inside each block are densely interconnected in the network, resulting in a regionally dense connectivity pattern that emphasizes the high-level properties created by the last layer for HSI classification. These approaches have shown that combining information from multiple layers in the CNN can improve HSI classification accuracy, but only a few fully leverage hierarchical features. Inspired by DenseNet architecture, the paper proposes a robust squeeze–excitation DenseNet network (SE-DenseNet) for HSI classification that makes full use of the characteristics acquired by each convolutional layer. Unlike DenseNet, which only creates dense connections within each block, the suggested solution connects any two layers in a feed-forward fashion across the whole network, resulting in fully dense connectivity. In this approach, features from previous layers are integrated as the current layer’s input, and the current layer’s output is supplied into subsequent layers, resulting in a maximum flow of information and feature reuse and recalibration. In addition, for HSI classification, all hierarchical features, including dynamical information, are merged to obtain more discriminative features.

The core building block of CNN is the convolution layer, which operates to gather an abstract feature by merging both spatial and spectral band information into deep channels. This is a very important operation where the relationship between each channel should be sensibly explored [32]. Further, for feature standardization, a squeeze and excitation (SE) structure is proposed to adhere to the interdependencies between the channels of convolution features [33]. The SE block works on two basic operations: squeeze and excitation. In the squeeze process, the CNN block is capable of mapping the channel interdependencies by accessing global information. Therefore, they are able to recalibrate the feature map along with their spatial dimensions, and the excitation process produces the per-channel weights for the squeezed output. SE can produce better features and drive the performance gains higher. These SE blocks can be combined into deep learning methods, such as dense networks. In [34], an improved CNN performance for classification was shown using SE-Net, which uses a global average pooled feature for gaining attention in channels. Moreover, in [33], they proposed a model using 3D-CNN with a discriminating spectral attention method for extracting spectral information and re-calibrating the spectral bands (MSDN-SA) for HSI classification. However, most of the research does not consider the spatial features of the HSI, so the combined information of spatial and spectral is not learned by the network.

Noise, instability, and redundancy are common features of raw input data that must be removed before proceeding to the major processing steps. In addition, input layers may have varying scales and dynamic ranges, which might have an impact on other levels. PCA can adapt the input HSI data into a space where the HSI data has the most variability in every axis by scaling. As a result, after normalizing the input HSI data, the PCA transformation is applied in our proposed classification approach. This paper develops an optimized SE dense network to excite or suppress features in the spatial-spectral of HSI, and the AdaBound optimizer is used to train the model with an extremely high learning rate. In addition, the overfitting problem is addressed using several regularization methods that are implemented for HSI classification. Among the most popular regularization techniques are L2 regularization, batch normalization (BN), and dropout [35,36]. In this letter, an efficient regularization technique termed “cutout” is used for HSI classification. The advantage of cutout is that it eliminates random square regions in the input layer and the back-propagation algorithm could help these regions spread, whereas dropout eliminates neuron cells in the transitional layer of CNN [37]. Instead of individual pixels, the proposed regularization method dynamically masks off a normal square from extracted features. Additionally, extracted feature cutout provides feature maps with multiresolution sizes and is simple to apply when compared with dropout. Because the cutout region is configured to be larger than 50% of the input, the region area may not always be fully engulfed within the convolution layer. As a result, feature maps with varying levels of region sizes are produced, and multiresolution feature cutout minimizes the overfitting problem even further. In this paper, a new standard for designing CNN architectures for HSI classification is carried out. Experiments on two publicly available hyperspectral images show that the proposed SE-AB-DenseNet with cutout outperforms various state-of-the-art techniques, particularly with limited training data. The main contributions to this paper are summarized as follows:

1. The SE-AB-DenseNet with cutout is developed and can be used to train the model to motivate or suppress features of the spectral bands or spatial dimensions. This helps to reduce the noise in spectral bands and pixel irregularity in the spatial surroundings.
2. The SE-AB-DenseNet with cutout consists of an AdaBound optimizer, which is used to train the classifier at an extremely high learning rate, such that the model can generalize fast and efficiently.
3. In order to address the issue of overfitting in the SE-AB-DenseNet model and, moreover, to improve the classification accuracy performance, the cutout regularization technique is incorporated.
4. The SE-AB-DenseNet with cutout was investigated at low training parameters on widely used hyperspectral datasets.

This paper is organized as follows: Section 2 introduces the detailed architecture of the proposed SE-AB-DenseNet with cutout. Experimental results are shown in Section 3. Section 4 summarizes the evaluation of the network’s performance attributes. Finally, Section 5 concludes the paper.

## 2. Datasets and Proposed SE-AB-DenseNet

This section introduces the SE-AB-DenseNet with cutout model as a spatial–spectral classifier and uses the AdaBound optimizer to train the classifier efficiently. Moreover, the cutout regularization technique is used to overcome the issue of overfitting.

### 2.1. Classification of Spatial–Spectral Information Using Squeeze–Excitation AdaBound Dense Network (SE-AB-DenseNet) with Cutout

The proposed SE-AB-DenseNet with cutout model can adapt to learn the weights of different spectral bands and different target pixels simultaneously. It is inspired by the re-calibration effect of the SE model. This characteristic of the model degrades the noise inference and enhances the classification performance.

### 2.2. Dense Network

**Figure 1:**
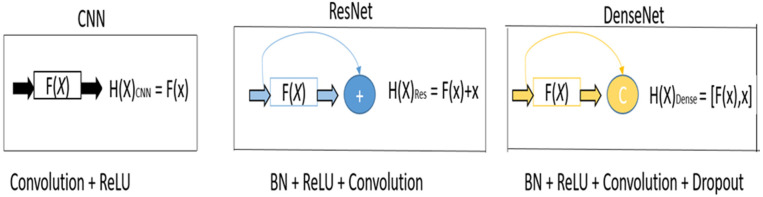
Basic design of CNN, ResNet, and DenseNet.

It is known that convolutional neural networks (CNN) are the best choice for extracting features and image processing applications. Usually, a traditional CNN consists of a convolution layer, pooling layers, fully-connected layers, and a prediction layer. However, if the number of layers in the CNN model were increased, better feature extraction could be done. However, increasing layers causes a vanishing gradient problem, and hence ResNet [38], also known as the residual approach, helped to solve the problem of vanishing gradient. In recent times, an advanced version of ResNet [32] was designed, where the training convergence of ResNet layers did not change, so redundancy increased as the input came from the previous layer output. Furthermore, DenseNet [33,34] was explored to reuse and recalibrate the extracted features, which eased computations, flexible training, and consistent parameter usage. Figure 1 shows the basic design scheme of CNN, ResNet, and DenseNet, in which the DenseNet present layers are the input for the next subsequent layer.

By reusing features, DenseNet extracts more significant properties, maximizing network efficiency. The extracted features retain both spatial and spectral information.

Optimum HSI classification can be achieved by adding spectral dimension to DenseNet’s convolutional and pooling layers. To further reduce the model parameters, an additional growth rate (r) is introduced into the layers, which reduces overfitting and saves computational resources.

### 2.3. Squeeze–Excitation (SE) DenseNet Block for HSI Classification

Squeeze–excitation DenseNet block uses a feature recalibration technique to generate a feature map from different convolutions that corresponds to the input feature. The advantage of using the SE block is that it can maximize the interdependency and enrich the essential information of the HSI classification. The SE block adapts and excites features at a very low level, while it enhances the features earlier. Henceforth, the whole network will be recalibrated using the SE block.

The spectral information in the SE block recalibrates the features on each level by modeling the complex interdependencies among the deep layers. Let us depict the input of the SE block, which represents the number of feature layers. It corresponds to the local region, hence there is a shortage of global information in it. To address this issue, the global receptive information is squeezed into the block descriptor. The global receptive operation on spatial dimesons helps to achieve the layer-wise feature.

The excitation operation helps the spatial–spectral feature generate weights. The parameters of the block are learned by the correlation feature among the layers. To generalize the model and reduce the complexity, two fully connected layers (FC) are used. The first FC layer is used to decrease the dimension and the second FC layer to return to the original dimension. By using two FC layers, the model’s linearity can be maintained and can achieve reduced parameters with fewer computations. To normalize the weights, the sigmoid function is used, which acts as a simple gating technique while capturing the features. Finally, the recalibration result is scaled using the ReLU activation value.

### 2.4. Structure of SE-AB-DenseNet for HSI Classification

The SE-AB-DenseNet model with cutout is a combination of DenseNet which is enhanced with SE blocks, cutout regularization approach, and an adaptive AdaBound optimizer. Moreover, spectral–spatial features are combined to obtain improvised classification accuracy.

The dense block is used to learn and categorize spatial–spectral features in different convolution layers. The DenseNet uses both the spectrum from the pixel as input and its patch to extract the feature. The proposed SE-AB-DenseNet with cutout model inherits DenseNet architecture characteristics such as reuse and recalibration, easing the vanishing gradient issue and optimizing model parameters followed by SE transformations. The dense block receives many appropriate spatial–spectral features, whereas SE helps them improve the quality of the obtained features. Cutout is being explored as a regularization approach to minimize the overfitting problem in SE-AB-DenseNet. Moreover, the use of the AdaBound optimizer helps the dense block to generalize efficiently. Hence, the proposed method allows the model to perform feature recalibration by which it can use the overall information features and suppress the unwanted ones. 

**Figure 2:**
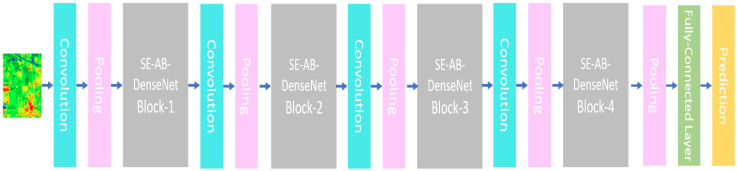
The basic structure of proposed SE-AB-DenseNet with cutout.

The block diagram of an SE-AB-DenseNet with a cutout model is illustrated in Figure 2. The model consists of four dense blocks and each has an SE module. The proposed model has 4 convolution layers, 5 pooling layers, and 2 fully connected layers. The hyper parameters are depicted in Table 1, and each dense block structure is shown in Figure 1.

A SE-AB-DenseNet block is comprised of a convolution layer and SE modules. A layer is added to the subsequent layers and its output is the input for the next layer. Meanwhile, the SE block is associated with a 3 × 3 convolution layer. Its structure consists of a global average pooling layer to obtain the global extraction of the feature maps. Later, it has fully-connected layers to obtain the weights from the layers. Lastly, the actual feature maps are recalibrated with new weights.

The design description of the proposed model is given in Table 1. The proposed model has four SE-AB-DenseNet blocks as shown in Figure 2. In the Indian Pines dataset, an initial 1 × 1 convolutional kernel is used to extract the feature. The IP dataset has dimensions of 11 × 11 × 200, which are compressed to 11 × 11 × 128 dimensions by carrying out a convolution operation with 128 filters of dimensions 1 × 1 × 200. The flexibility between blocks and channels is a great advantage in this model. After the blocks are structured, feature maps are compiled into one-dimensional vectors using global pooling. Lastly, Softmax is utilized to estimate the prediction labels of the corresponding classes.

It is essential to select the appropriate optimizer to improve the deep neural network results. The most commonly used optimizer is stochastic gradient descent, which has a high degree of generalization, however, generalization alone is insufficient. To achieve a high convergence rate, adaptive optimizers such as Adam, AdaDelta, AdaGrad, and RMSprop are required. However, these adaptive optimizers need high learning rates [36] and their ability to generalize is low. Hence, the AdaBound optimizer is selected to improve the training process with extraordinary generalization capability and convergence. The role of the AdaBound optimizer is briefed below [36].

#### 2.4.1. AdaBound Optimizer in SE-AB-DenseNet Model

The SE-AB-DenseNet model is trained with the AdaBound optimizer. The AdaBound optimizer uses the dynamic constraints on learning rates to achieve the objective of transitioning from an adaptive to an SGD optimizer, which reduces the generalization gap between adaptive and SGD approaches but also keeps the learning rate higher in the initial stages of training. The steps below show the basics of the AdaBound optimizer [33].

Step 1: Input the initial element step size as *σ*, ${\left\{\alpha_{1t}\right\}}_{t=1}^{n}$, $\alpha_{2}$ and bound function is given by $\varepsilon_{l}\;\mathrm{and}\;\varepsilon_{u}$ (both lower and upper bound)

$$\varepsilon_{l}\left(t\right)=0.1-0.1/((1-\alpha_{2})*t+1)\;\varepsilon_{u}\left(t\right)=0.1-0.1/((1-\alpha_{2})*t)$$

Step 2: Set the vector values *m* and *n* to 0

Step 3: $gradient_{t}=\nabla l_{t}(w_{t})$

The gradient function ∇ of the *t*th iteration is obtained with $w_{t}$ parameters and $l_{t}$ loss function.

Step 4: computing $m_{t}\;\mathrm{and}\;n_{t}$

$$m_{t}=\alpha_{1t}*m_{t-1}+(1-\alpha_{1t})*gradient_{t}\;n_{t}=\alpha_{2}*m_{t-1}+(1-\alpha_{2})*gradient_{t}^{2}\;D_{t}=diag\left(d_{t}\right)$$

Step 5: Repeat from step 2 to step 5 after updating the parameter following

$$\varepsilon_{t}^{\prime }=clip\left(\frac{\sigma }{\sqrt{D_{t}}},\varepsilon_{l},\varepsilon_{u}\right)\;\varepsilon_{t}=\varepsilon_{t}^{\prime }\sqrt{t}$$

where clipping on learning rates converges asymptotically.

$$clip\left(\frac{\sigma }{\sqrt{D_{t}}},\varepsilon_{l},\varepsilon_{u}\right)=\left\{\begin{array}{c} \frac{\sigma }{\sqrt{D_{t}}}\frac{\sigma }{\sqrt{D_{t}}}\epsilon \left[\varepsilon_{l}\left(t\right),\varepsilon_{u}\left(t\right)\right]\\ \varepsilon_{l}\left(t\right)\frac{\sigma }{\sqrt{D_{t}}}<\varepsilon_{l}\left(t\right)\\ \varepsilon_{u}\left(t\right)\frac{\sigma }{\sqrt{D_{t}}}>\varepsilon_{u}\left(t\right) \end{array}\right\}\;w_{t+1}=\arg\;min{\;}_{w\epsilon L}({\left(diag\left(\varepsilon_{t}^{-1}\right)\right)}^{\frac{1}{2}}\left(w-\left(w_{t}-\varepsilon_{t}⊙m_{t}\right)\right)$$

In the above formulae, the learning rate can be transformed as a function of t, and the variation between the upper and lower limits of the learning rate will decrease, causing the proposed optimizer to behave like Adam at first because the bounds have little effect on learning rates, and then gradually transform into SGD as the bounds become more confined [36].

#### 2.4.2. Regularization Using Cutout

Deep learning-based HSI classification approaches usually face a major overfitting problem owing to the increased dimensionality of HSI inputs combined with a huge number of training examples in deep learning models. Overfitting occurs when models fail to generalize, i.e., although the training error is less, the test error is excessive [39]. When the amount of training data is small, overfitting is significantly more serious in the HSI classification. To avoid overfitting, deep learning-based HSI classification algorithms require a good regularization strategy. Cutout regularization is a unique and efficient regularization strategy for CNN-based HSI classification. Dropout is a common strategy for dealing with overfitting that is widely employed in several research domains, including HSI classification [40]. In this paper, with minimum training data, the cutout technique is paired with dropout to further reduce overfitting issues in the HSI classification.

**Figure 3:**
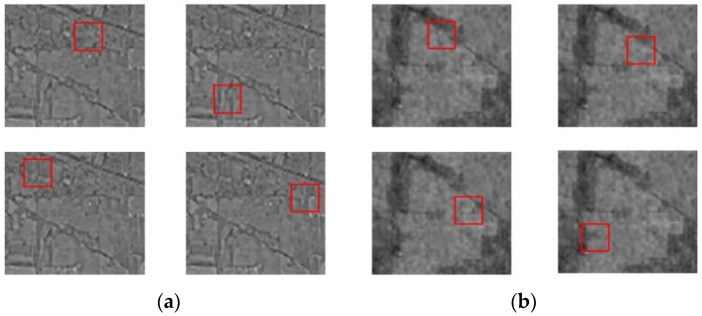
Cutout regularization on Indian Pines dataset with two examples: (**a**) example 1 and (**b**) example 2.

Cutout regularization removes sections randomly from the input layer rather than the feature layers, and conceals the input features with continuous adjacent pixels rather than subpixels. Furthermore, the cutout is a simple approach to implement. Algorithm 1 shows the overall cutout process for HSI classification in SE-AB-DenseNet with cutout. Cutout arbitrarily removes the overlapping sections of the bands that are randomly selected for HSI classification. The Figure 3 depicts the cutout approach used on the HSI Indian Pines data set, which shows the outcomes of the cutout operation on the same scenario at several bands and locations.
**Algorithm 1:** SE-AB-DenseNet with Cutout for HSI Spatial–Spectral Classification **Input: Start** **Step 1**: Set for each pixel, neighborhood size **N**, number of training sample data **Ts**, validation sample data **Vs**, and an operation variable **O**. **Step 2**: Resize the input to **N × N** for all neighboring space of each pixel and make a sample set. **Step 3**: Perform a split on sample set as training set, validation set, and test set similar to **Ts** and **Vs**. **Step 4**: Proposed model search:  Set learning rates and ε, weights ω, model variable ϑ, number of cutout band $N_{b}$, cutout length $L_{c}$, and epochs.   For every sample in training set.   Arbitrarily select bands $N_{b}$     For each $N_{b}$:      Set $L_{c}$
**×**
$L_{c}$
**= 0** for all pixels    For every epoch perform:     $\omega =\omega -\rho \nabla_{\omega }L_{c-train}\left(\omega ,\vartheta \right)$
    $\;\vartheta =\vartheta -\varepsilon \nabla_{\omega }L_{C-val}\left(\rho \nabla_{\omega }L_{c-train}\left(\omega ,\vartheta \right),\vartheta \right)$     Opt for the finest “ϑ” as it performs on validation set. **Step 5**: Train and test the dataset on proposed model  Set weights ω of newly formed trained dataset, learning rates ρ and epochs.     For each epoch:      For every batch size:     $\;\omega^{\prime }=\omega^{\prime }-\rho \nabla_{\omega }*L_{c-train}\left(\omega^{\prime }\right)$     Predict for every test of batch size   Compute overall accuracy (**OA**), average accuracy (**AA**), **and** kappa-coefficient (**k**) depending on prediction and test labels **End**

### 2.5. Structure of Spatial–Spectral Squeeze and Excitation Block

The squeeze and excitation blocks transform the dimensions of spectral features, and spatial feature maps are compressed to extract the maximum information. This interdependence information in the SE block helps in obtaining the global information from the HSI classifications. 

Spectral: In the spectral SE block, the spatial feature maps are squeezed and spectral features are excited. Let $X=\{x_{1},x_{2},\ldots .x_{n}\}$ be the input to the SE block and provide the information from the low level of the channel [20]. The squeeze operation of global information is given by $q\epsilon C^{n}$

(1) $$q_{c}=S_{sq}\left(x_{c}\right)=\frac{1}{P\times Q}\overset{P}{\underset{i=1}{\sum }}\overset{Q}{\underset{j=1}{\sum }}x_{c}\left(i,j\right)c=1\ldots \ldots n,$$

where P×Q is the feature map dimensions of *C*th channel $x_{n}\epsilon C^{P\times Q}$, *i* and *j* are subsequent feature map inputs for SE block, and $S_{sq}\left(.\right)$ is squeeze operator.

To the squeezed information, the excitation is applied through a sigmoid activation function *σ*. The excited feature is valued as:

(2) $$e=S_{ex}\left(q,W\right)=\sigma \left(W_{1}\left(\delta \left(W_{2}q\right)\right)\right),$$

where $S_{ex}$ is the excitation feature to obtain the final stimulation value, δ is the ReLU function, $W_{1}\;\mathrm{and}\;W_{2}$ are the weight matrices of two-fully connected layers that help in reducing the complexity of the model.

The output of the squeeze excitation block after the operation is:

(3) $$X_{Spectral}=\left\{e_{1}x_{1},e_{2}x_{2},\ldots .e_{n}x_{n}\right\}$$

Spatial: In the spatial SE block, the *X* feature maps are squeezed and compressed to adhere the information from all channels of HSI classification. Let the spatial dimension be excited by $X=\left\{x^{1,1},x^{1,2},\ldots x^{i,j},\ldots ,x^{P,Q}\right\}$ and the dimensions are reduced to 1 × 1 × *n* with feature position at (*i*, *j*) [20]. Here, convolution and sigmoid function are used to perform the squeeze excitation operation:

(4) $$t=S_{ex}\left(S_{sq}\left(X\right)\right)=\sigma \left(P⊗X\right)$$

where $t^{i,j}$ represents the excited state of all channels in *X* at positions (*i*, *j*)

(5) $$X_{spatial}=\left\{t^{1,1}x^{1,1},\ldots \ldots t^{i,j}x^{i,j},\ldots t^{p,q}x^{p,q}\right\}$$

$X_{spatial},$ output for spatial excitation features by multiplying *X* input with extracted activations.

#### Spatial–Spectral Squeeze–Excitation AdaBound DenseNet (SE-AB-DenseNet) Classifier 

**Figure 4:**
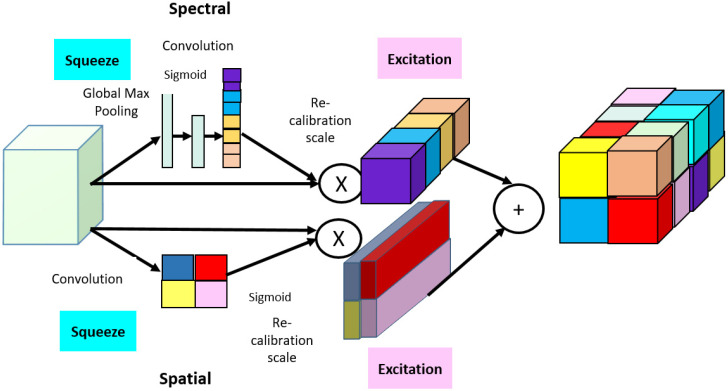
Spatial–spectral squeeze-and-excitation block structure.

**Figure 5:**
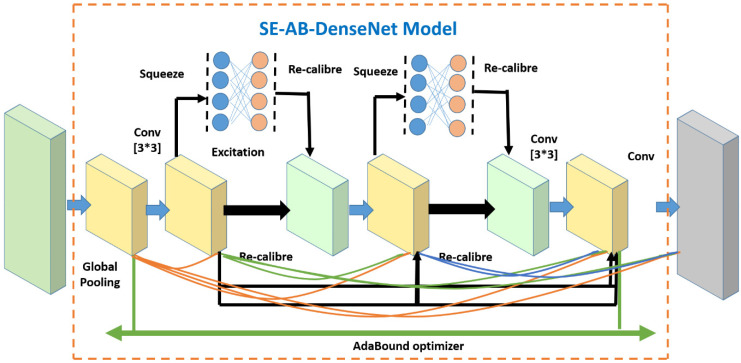
Proposed overall block diagram of SE-AB-DenseNet with cutout classifier.

The spatial–spectral squeeze–excitation structure is given by

(6) $$X_{SE}=\theta \cdot X_{Spectral}+\left(1-\theta \right)\cdot X_{spatial}$$

where θ is a variable that requires to be trained for both spatial and spectral excitation and the activation value will be high while computing the spatial dimension (*i*, *j*, *n*). This computation inspires the model to acquire more relevant information from the feature map. Figure 4 shows the structure of the spatial–spectral squeeze-and-excitation AdaBound DenseNet classifier and Figure 5 illustrates the overall proposed block diagram of SE-AB-DenseNet with cutout classifier.

### 2.6. Datasets

**Figure 6:**
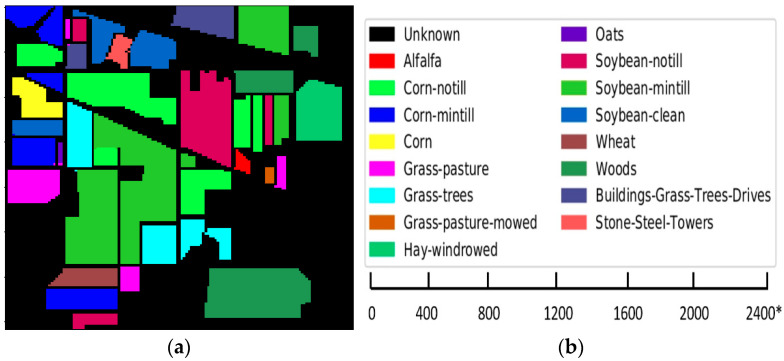
(**a**) Indian Pines dataset map; (**b**) color codes with scale bar (* is approximate scale of dataset).

The two benchmark datasets are used to evaluate the proposed model:

1 Indian Pines (IP): an airborne visible-infrared imaging spectrometer (AVIRIS) sensor captured a hyperspectral image of Indian Pines. It has 145 × 145 pixels and 220 spectral bands of wavelength ranging from 0.4–2.5 µm. Here, 20 bands were removed as they were affected by the atmospheric disturbances. The IP data has a spatial resolution of 20 m and 16 classes. Figure 6a Shows Indian Pines map and Figure 6b gives the scale bar of Indian Pines dataset. Table 2 show classes and their samples, respectively.

**Figure 7:**
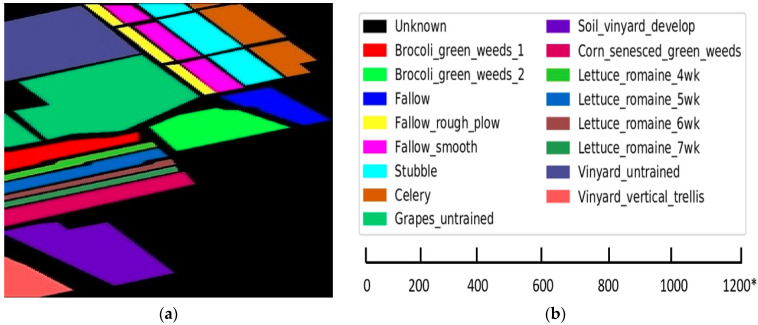
(**a**) Salinas dataset map; (**b**) color codes with scale bar (* is approximate scale of dataset).

2 Salinas (SA): the Salinas dataset has 224 bands and was collected by the AVIRIS sensor. The Salinas dataset is from the Salinas Valley, California, and has a spatial resolution of 3.7 m. In the SA dataset, 20 bands are removed as they are disturbed by water absorption. It includes vegetables, bare soil, and vineyard fields. Figure 7a shows Salinas map and Figure 7b gives the scale bar of Salinas dataset. Table 3 show the Salinas image with 16 classes.

## 3. Experiment and Results 

In the CNN input, the best optimal window size of 9 × 9 is chosen for both the dataset and the CNN input to balance the spatial information and its computation cost. The finest learning rates for IP and SA datasets are 0.0003 and 0.0006, respectively, and $1\times 10^{-5}$ is the weight decay for the model. The batch size is 32, and the model is trained for 100 epochs on each dataset. Of the total training samples, 90% were used to train the parameter and 10% were used as a validation set. A standard metric to compare the performance of different techniques is used to assess the performance. The overall accuracy (OA), average accuracy (AA), and kappa coefficient (k) are documented on the testing set, and the results are shown in Table 4 and Table 5, respectively. Table 4 and Table 5 displays the best accuracy of distinct classes obtained in each classifier (bold highlighted results). 

## 4. Classification Results

To substantiate the overall performance of the proposed SE-AB-DenseNet method, it was compared with SVM [42], 2D-CNN [26], 3D-CNN [24], spectral–spatial ResNet SSRN [23], HybridSN [27], and DPSCN (dual-path small convolution network) [43]. It is observed that:

- The proposed SE-AB-DenseNet with the cutout model delivers the best classification accuracy results on the IP and SA datasets.
- The cutout is a regularization approach used in order to alleviate the overfitting problem in a proposed model and boost classification performance further. The combined use of spatial and spectral information has shown improved results in spectra–spatial-based approaches.
- The AdaBound optimizer provided the network with the ability to improve its stability and enhance its classification accuracy by approximately 2% with the SSRN and DPSCN methods with the Adam optimizer. While with HybridSN method improved the accuracy by 0.30% for the Indian Pines dataset in Table 4.
- Furthermore, the application of the AdaBound optimizer in the proposed method improved the classification accuracy by 2% for both datasets, as shown in Table 4 and Table 5. The model’s classification accuracy is compared with and without the AdaBound optimizer in it.
- The proposed SE-AB-DenseNet with the cutout model can extract spectral–spatial features efficiently by specifying the important spectral bands and avoids overfitting of spatial–spectral information.

**Figure 8:**
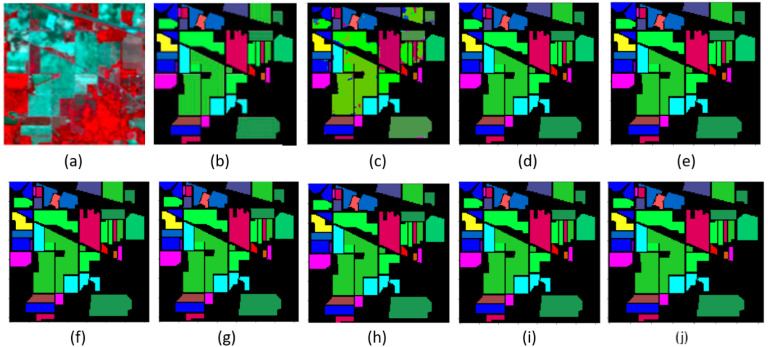
Indian Pines classification maps: (**a**) RGB (bands 32, 24, and 11) image; (**b**) ground truth; (**c**) SVM; (**d**) 2-D CNN; (**e**) 3-DCNN; (**f**) SSRN; (**g**) HybridSN; (**h**) DPSCN; (**i**) SE-DenseNet with cutout; and (**j**) SE-AB-DenseNet with cutout.

**Figure 9:**
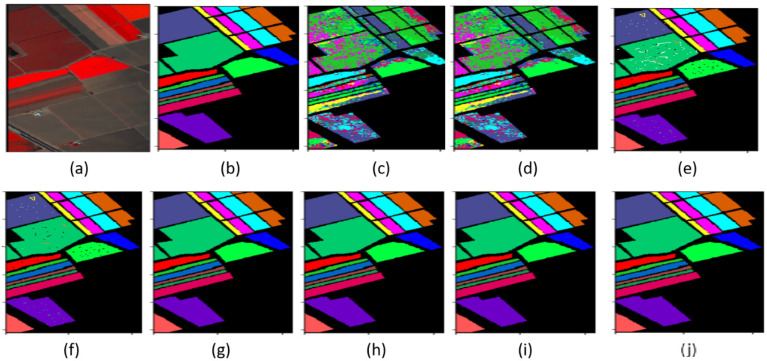
Salinas classification maps: (**a**) RGB (bands 57, 19, and 9) image; (**b**) ground truth; (**c**) SVM; (**d**) 2-D CNN; (**e**) 3-DCNN; (**f**) SSRN; (**g**) HybridSN; (**h**) DPSCN; (**i**) SE-DenseNet with cutout; and (**j**) SE-AB-DenseNet with cutout.

The classification maps of the Indian Pines and Salinas datasets are shown in Figure 8 and Figure 9, with different classifiers such as SVM, 2D-CNN, 3D-CNN, R-SSRN, HybridSN, DPSCN, SE-DenseNet with cutout, and proposed SE-AB-DenseNet with cutout accuracies, respectively. The proposed robust model SE-AB-DenseNet with cutout extracted deep features with interclass firmness and shows nearly ~2% of improved accuracy in both datasets. This spatial–spectral extraction together has provided the features with much better clarity on class centers. The classified map obtained is very similar to the ground truth and the pixels are classified better. Whereas, SVM is a spectral-based classifier that generates a noisy classification as it collects only the remote spectral samples and spatial neighboring feature information is not used. The 3D-CNN and R-SSRN are spatial–spectral-based classifiers that provide better classification than spectral-based classifiers with clear boundary areas. In both datasets, the HybridSN model almost matched the accuracy of the SE-AB-DenseNet with the cutout model. DPSCN, on the other hand, outperforms 2D networks but achieves lower accuracy than SSRN and HybridSN. The SE-AB-DenseNet model consistently outperformed traditional techniques, as the SE blocks learned spectral representations that are related to spatial information. Despite the fact that there are few training examples for alfalfa, oats, and grass-pasture classes, the SE-AB-DenseNet model identified the testing data in the Indian pines dataset with greater than 98% accuracy in their classification The sustainability of the proposed method is demonstrated by these results. In the face of adversity, the intended models perform admirably. The proposed SE-AB-DenseNet with cutout model, in particular, is more effective when using a squeeze–excitation block, AdaBound optimizers, and effective cutout regularization parameters.

**Figure 10:**
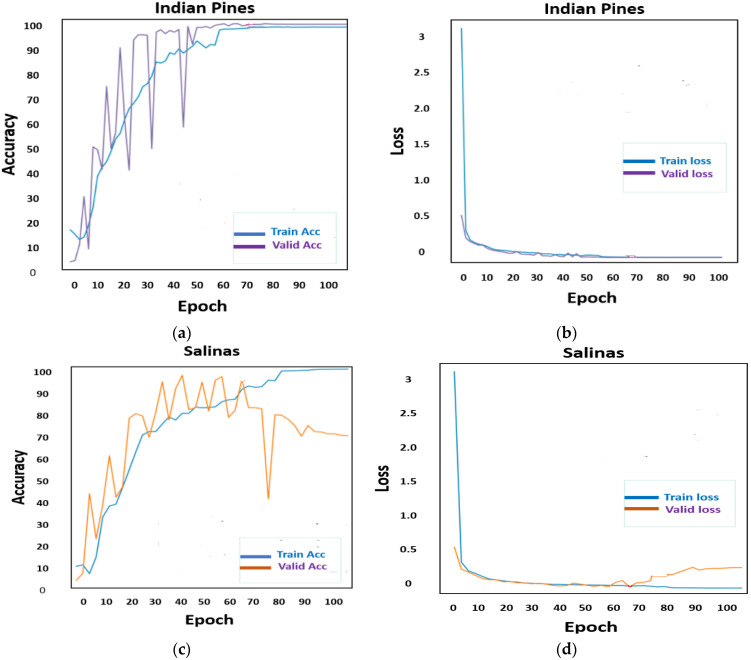
Learning curves for Indian Pines (IP) and Salinas (SA) datasets with cutout regularization: (**a**) accuracy of IP; (**b**) loss of IP; (**c**) accuracy of SA; and (**d**) loss of SA.

For two key reasons, the SE-AB-DenseNet with cutout obtained improved classification performance with a smaller number of trainable samples. First, when the number of training samples is restricted, a model with a large number of trainable parameters tends to overfit. Second, the SE-AB-DenseNet with cutout is designed automatically using the training and validation data. This improves the classification accuracy even further. Figure 10 shows the training and validation accuracy and loss plot for the proposed model with cutout regularizations on both datasets.

### 4.1. Evaluation of Network Parameters for Improving the HSI Classification

**Figure 11:**
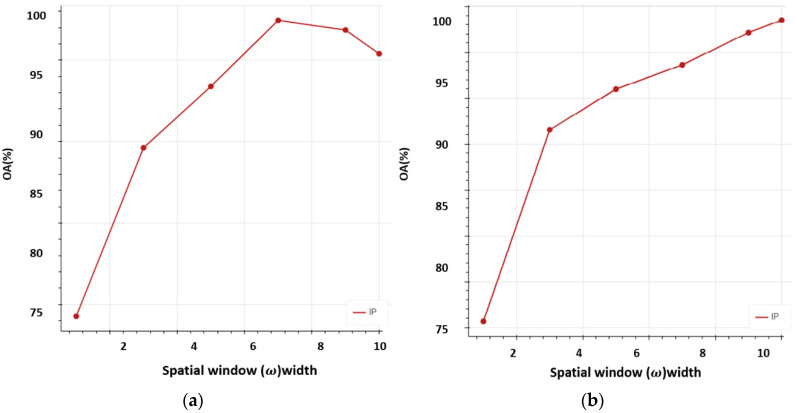
Shows the overall accuracy (OA%) vs. spatial window (ω) width: (**a**) 5% training samples; (**b**) 10% training samples.

This section deals with the effects of different parameters on the proposed network. The width of the input window, along with the trainable coefficient, is varied with the ratio of spatial–spectral squeeze excitation blocks [44]. Window width controls the size of the input features and SE blocks depth. A different percentage of the training set samples for each class from the IP and SA datasets are used. Firstly, different window sizes (3, 5, 7, and 9) are used and their corresponding OA values of SE-AB-DenseNet with cutout are plotted for 5% and 10% of the training samples, shown in Figure 11. It is observed that the OA value exponentially increases with the increase in window size (9), obtaining the maximum value. Hence, window size 9 is used in carrying out the experiments.

Then, the trainable variable effect is observed in Figure 11. Equation (6) shows how the SE-AB-DenseNet model performs as a spatial SE model and how it performs as a spectral SE model. Correspondingly, both the spatial and spectral SE blocks have a balanced effect on the proposed model. Figure 11 shows the influence on OA of SE-AB-DenseNet with cutout, respectively.

**Figure 12:**
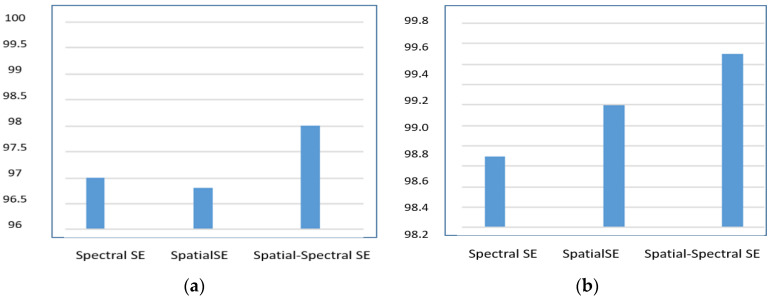
The trainable coefficient θ vs. overall accuracy (OA%): (**a**) 5% training samples; (**b**) 10% training samples.

Additionally, in the SE-AB-DenseNet with cutout model, the efficacy of the spectral–spatial SE block is inspected with trainable co-efficient θ. When θ = 1, the model acts as a spectral SE block; when θ = 0, the model acts as a spatial SE; and when θ = 0.5, it acts as a spatial–spectral SE block. Figure 12 shows the OA of the proposed model with different θ values. It is observed that the spatial–spectral SE combined block provides the better results. 

**Figure 13:**
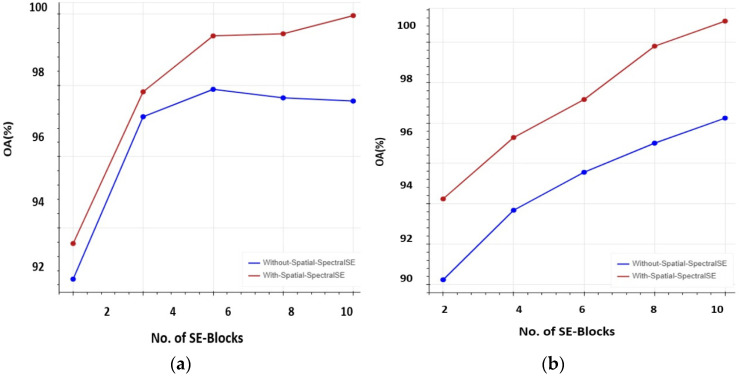
With and without spatial–spectral SE blocks vs. overall accuracy (OA%): (**a**) 5% training samples; (**b**) 10% training samples.

Moreover, the evaluation with and without SE blocks were carried out on the proposed model. When SE blocks are removed from the proposed model, it acts as a simple dense network. The OA of the proposed model with and without SE blocks is shown in Figure 13. It is very clear from the graph that SE blocks add more effectiveness than traditional models and three or four is the average number of spatial–spectral SE blocks to be used.

### 4.2. Exploring Spatial–Spectral Effects on Class Samples

Furthermore, the usefulness of the spatial–spectral SE block in the proposed model, SE-AB-DenseNet with cutout, was observed to determine how the model improved its performance. Moreover, it is important to identify the practical instinctive mechanism of the SE-AB-DenseNet with the cutout model. Hence, in this section, classification features of discrete samples’ behaviors are observed in the proposed model, and different classes of different SE blocks are also studied. For experiments, the Indian Pines dataset is considered as it focuses on strong vegetation classes; however, among them, randomly, there are four different classes (class 1, class 3, class 5, and class 11) and 20 samples are chosen for each to compute the average behavior of spatial–spectral blocks in different layers of the model.

**Figure 14:**
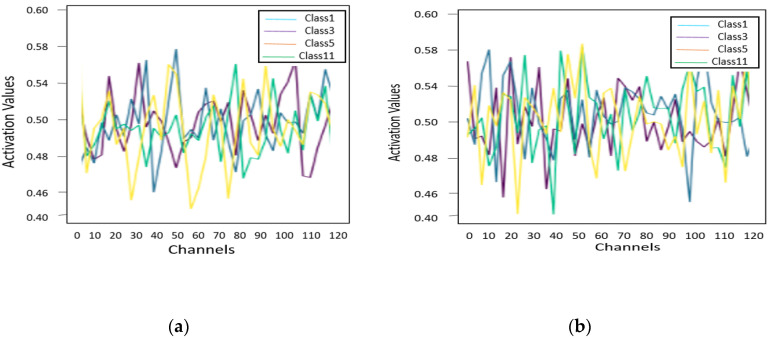

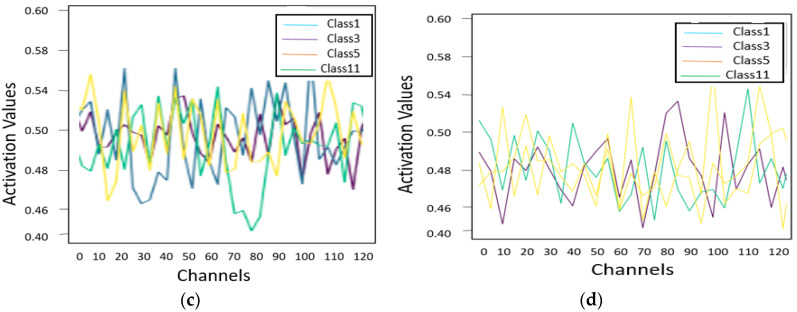
Illustration of the spectral behavior of four different classes in SE blocks: (**a**) SE-1; (**b**) SE-2; (**c**) SE-3; and (**d**) SE-4.

In considering both features, spatial and spectral visualizations are observed separately. Figure 14 shows the composite spectral dimension activation values for the selected classes. It can be seen that each class has a different activation value for each channel of the SE block. In Figure 14b, it is observed that classes 1, 3, 5, and 11 have a uniform compression effect at the 50th channel, stating that similar spectral behavior was observed in samples in each class.

**Figure 15:**
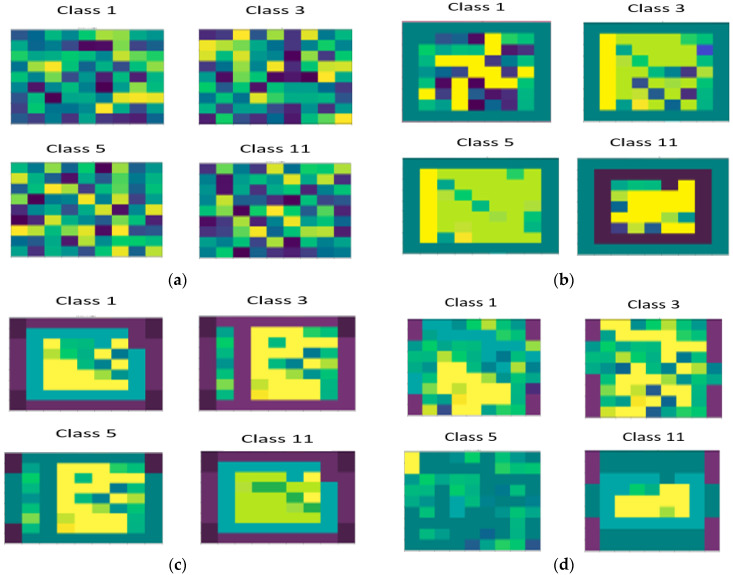
Illustration of the spatial behavior of four different classes in SE blocks: (**a**) SE-1; (**b**) SE-2; (**c**) SE-3; and (**d**) SE-4.

Meanwhile, in Figure 15, the spatial dimensions of four classes with different activation values on individual samples are examined and their prevalence of different classes throughout different SE blocks is evaluated. It is seen from the form figure that the gloomier part has the greater activation value. It is observed that at the center, the features are actively activated while at the boundaries, pixels are compressed. As the boundary pixels are away from the center pixel, the SE-AB-DenseNet with cutout model archives much improved performance results.

### 4.3. Discussion

**Figure 16:**
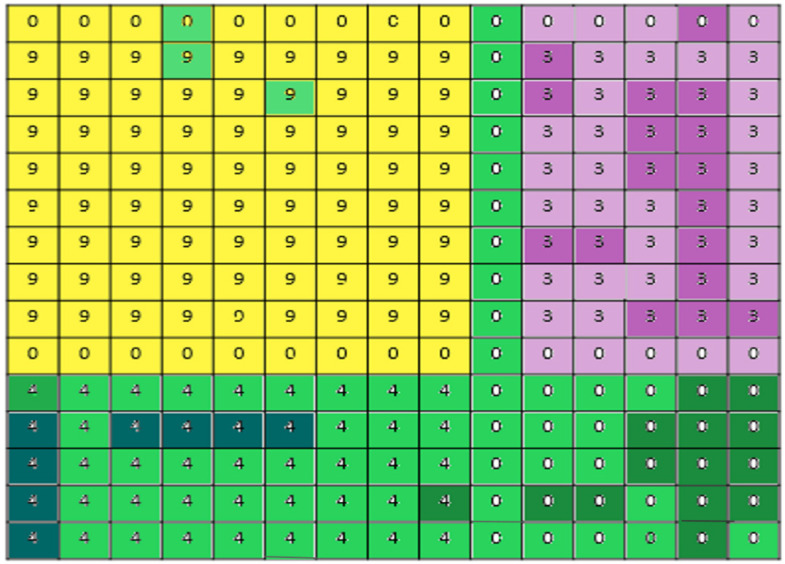
Spatial neighborhood representation of SE block after classification.

The SE model is used to recalibrate the spatial and spectral features with different algorithms to obtain better feature classification. Finally, the visualization effect of the proposed model is discussed in this section. From the Indian Pines dataset, a pixel from class 9 is selected and a 9 × 9 spatial window size is shown in Figure 16. It is seen that the pixels from class 9 are surrounded by 0 labeled pixels and pixels from classes 3 and 4. The effect of color shows the stronger and lighter activation or excited values. Hence, on computing spatial features, the similar pixels are masked and help in classifying the corresponding class. The other class pixels are compressed by not hampering the required classification.

The results of the experiments show that the SE-AB-DenseNet with cutout model is effective. It is worth mentioning that different deep learning models favor distinct hyperparameters, which makes implementing these models difficult. The experiment results show that the SE-AB-DenseNet with cutout model classification performance with various settings is steady. The SE-AB-DenseNet model has three primary factors: first, the SE-AB-DenseNet uses dense connections, which improve classification accuracy while also making deep learning models easier to train. Second, the SE-AB-DenseNet employs squeeze excitation blocks to address spectral and spatial variables separately in two blocks, allowing for the extraction of additional discriminative features. Third, feature maps with varying levels of region sizes are formed as a result of the cutout regularization process at each convolutional layer, and multiresolution feature cutout minimizes the overfitting problem.

This research work also performs admirably when only a small percentage of the training data is used. Table 4 and Table 5 show the outcomes of the experiment, in which both datasets achieve the best level of accuracy for the unusual training data The robustness of the proposed model is confirmed by these findings.

## 5. Conclusions

This paper constitutes a spatial–spectral squeeze-and-excitation AdaBound dense network (SE-AB-DenseNet) with a cutout model for HSI classification. The SE-AB-DenseNet model achieves an improvised classification accuracy when compared with existing models comprising SVM, 2D-CNN, 3D-CNN, R-SSRN, HybridSN, and DPSCN. The special nature of deep learning models presents the input data automatically. Furthermore, the number of training samples and the spatial dimension of each sample influence the hyper-parameter settings. One significant problem in HSI classification is the scarcity of labels. As a result, this work proposes a spectral–spatial squeeze–excitation DenseNet architecture that considers both numerous spectral and spatial information contexts. It is important to mention that this model has been able to obtain reliable classification results with both small and large amounts of unequal training data. In the dense network framework, the proposed SE-AB-DenseNet model has four SE blocks, which excite and compress features of spatial and spectral dimensions, respectively. The recalibrated feature improves the performance of the proposed model. The optimizer, AdaBound, helps the proposed model to improve classification accuracy faster. It is used in the design of the proposed model and an improved result of nearly 2% is achieved for HSI classification. The cutout regularization approach used in order to alleviate the overfitting problem and improvised result is obtained. The benchmark datasets Indian Pines and Salinas showed remarkable results for classification using the SE-AB-DenseNet with cutout model. Finally, for its consistent structure and deep feature learning potential, the proposed SE-AB-DenseNet model achieved state-of-the-art results with limited labelled data and can effectively be applied to various remote-sensing applications.

## Figures and Tables

**Table 1:** Structure details of SE-AB-DenseNet network.

Network Layers	Kernel Size	Details of Parameters
Input	200*200	Conv-1*1, 200, 128
Convolution (Conv-1)	112*112	Stride 2
Pooling (Global)	56*56	Pooling-3 × 3, Stride 2
SE-AB-DenseNet Blk-1	56*56	[1 × 1, 3 × 3 conv (SE block-1)]*6
Convolution (Conv-2)	56*56	Conv-1 × 1
Pooling (Global)	28*28	Pooling-2 × 2, Stride 2
SE-AB-DenseNet Blk-2	28*28	[1 × 1, 3 × 3 conv (SE block-2)]*12
Convolution (Conv-3)	28*28	Conv-1 × 1
Pooling (Global)	14*14	Pooling-2 × 2, Stride 2
SE-AB-DenseNet Blk-3	14*14	[1 × 1, 3 × 3 conv (SE block-3)]*48
Convolution (Conv-4)	14*14	Conv-1 × 1
Pooling (Global)	7*7	Pooling-2 × 2, Stride 2
SE-AB-DenseNet Blk-4	7*7	[1 × 1, 3 × 3 conv (SE block-3)]*32
Pooling (Global)	1*1	Global pooling-7 × 7
Classification result	3*1	Layer-fully connected

**Table 2:** Training and test samples of Indian Pines dataset.

Class Number	Class Name	Total No. of Samples	No. of Training Samples (10%)	No. of Test Samples (90%)
1.	Alfalfa	46	5	41
2.	Corn-not ill	1428	143	1285
3.	Corn-mintill	830	83	747
4.	Corn	237	24	213
5.	Grass-pasture	483	49	434
6.	Grass-trees	730	73	657
7.	Grasspasture-mowed	28	3	25
8.	Hay-windrowed	478	48	430
9.	Oats	20	2	18
10.	Soybean-notill	972	98	874
11.	Soybean-mintill	2455	246	2209
12.	Soybean-clean	593	60	533
13.	Wheat	205	21	184
14.	Woods	1265	127	1138
15.	Buildings-Grass-Trees-Drives	386	39	347
16.	Stone-Steel-Towers	93	10	83
	TOTAL	10,249	1031	9218

**Table 3:** Training and test samples of Salinas dataset.

Class Number	Class Name	Total No. of Samples	No. of Training Samples (10%)	No. of Test Samples (90%)
1.	Brocoli_ green_weeds_1	1977	101	1908
2.	Brocoli _green_weeds_2	3726	187	3539
3.	Fallow	1976	99	1877
4.	Fallow_ rough_plow	1394	70	1324
5.	Fallow_smooth	2678	134	2544
6.	Stubble	3959	198	3761
7.	Celery	3579	179	3400
8.	Grapes_ untrained	11,213	564	10,707
9.	Soil_vinyard_ develop	6197	311	5892
10.	Corn_senesced_ green_weeds	3249	164	3114
11.	Lettuce_ romaine_4wk	1058	54	1014
12.	Lettuce_ romaine_5wk	1908	97	1830
13.	Lettuce_ romaine_6wk	909	46	870
14.	Lettuce_ romaine_7wk	1061	54	1016
15.	Vinyard_ untrained	7164	364	6904
16.	Vinyard_ vertical_trellis	1737	91	1716
	Total		2713	51,416

The Indian Pines (IP) and the Salinas (SA) datasets can be freely downloaded [41] at http://lesun.weebly.com/hyperspectral-data-set.html, (accessed on 17 June 2021).

**Table 4:** Class-wise overall accuracy (OA%), average accuracy (AA%), and *ĸ* kappa are represented for the Indian Pines dataset. This table displays the best accuracy of distinct classes obtained in each classifier (bold highlighted results).

Class	SVM	2D CNN	3D-CNN	SSRN	HybridSN	DPSCN	SE-DenseNet with Cutout	SE-AB-DenseNet with Cutout
Alfalfa	84.69	71.15	94.76	98.26	**99.45**	99.20	94.40	**99.02**
Corn-not ill	82.13	72.22	95.78	97.28	94.53	96.46	96.57	**98.78**
Corn-mintill	73.45	75.13	96.93	96.61	97.50	96.66	92.29	**97.53**
Corn	66.47	87.01	88.96	88.23	99.88	99.94	98.62	**100.00**
Grass-pasture	92.13	69.92	97.94	**98.37**	**99.16**	**98.28**	95.80	97.88
Grass-trees	97.38	93.43	96.89	**100.00**	89.46	99.66	99.05	**100.00**
Grasspasture-mowed	81.83	64.44	98.20	**99.02**	**100.00**	**100.00**	97.59	98.42
Hay-windrowed	97.89	98.13	99.29	95.46	95.80	**100.00**	91.68	**96.31**
Oats	71.74	83.73	77.81	94.68	94.67	**100.00**	96.40	**98.97**
Soybean-notill	73.61	77.89	97.50	99.19	96.75	94.84	96.35	**100.00**
Soybean-mintill	81.27	85.24	98.10	98.94	98.13	93.42	99.73	**100.00**
Soybean-clean	76.83	74.34	100.00	**100.00**	99.00	97.76	91.03	97.05
Wheat	97.01	98.72	98.03	94.85	**100.00**	**100.00**	89.49	**95.61**
Woods	93.36	94.17	99.28	**97.54**	99.38	98.24	93.85	96.84
Buildings-Grass- Trees-Drives	74.11	81.87	89.63	**89.29**	90.18	99.97	82.96	88.68
Stone-Steel-Towers	93.62	77.18	93.14	99.30	89.73	99.80	92.63	**99.55**
OA(%)	83.46	84.05	96.83	98.91	99.05	96.57	97.71	**99.37**
AA(%)	83.51	81.79	96.17	97.70	98.91	98.39	97.89	**99.08**
ĸ × 100	81.02	81.26	95.79	97.86	98.63	96.50	96.99	**99.26**

**Table 5:** Class-wise overall accuracy (OA%), average accuracy (AA%), and *ĸ* kappa are represented for the Salinas dataset. This table displays the best accuracy of distinct classes obtained in each classifier (bold highlighted results).

Class	SVM	2D-CNN	3D-CNN	SSRN	HybridSN	DPSCN	SE-DenseNet with Cutout	SE-AB-Dense Net with Cutout
Brocoli_ green_weeds_1	98.97	96.37	98.21	98.21	98.49	98.11	97.95	**99.23**
Brocoli _green_weeds_2	94.75	98.63	96.68	95.79	99.35	98.64	96.32	**99.87**
Fallow	91.11	86.50	88.91	92.62	98.41	98.03	96.51	**98.41**
Fallow_ rough_plow	97.21	98.41	97.37	96.51	98.60	96.92	95.69	**97.68**
Fallow_smooth	91.03	85.36	92.06	95.30	**100.00**	99.38	98.05	**100.00**
Stubble	87.51	97.74	98.15	89.77	99.57	**100.00**	97.94	**100.00**
Celery	92.65	95.42	99.02	97.21	99.56	98.86	96.79	**98.83**
Grapes_ untrained	89.91	96.25	75.24	92.45	99.85	86.72	94.68	**97.45**
Soil_vinyard_ develop	97.25	97.71	98.48	**98.58**	96.54	97.83	94.68	98.37
Corn_senesced_ green_weeds	74.25	77.34	77.95	88.48	97.45	99.04	93.79	**99.14**
Lettuce_ romaine_4wk	96.01	84.90	79.09	97.02	99.91	**100.00**	99.03	**100.00**
Lettuce_ romaine_5wk	98.19	98.28	97.67	**100.00**	**100.00**	99.56	97.49	**100.00**
Lettuce_ romaine_6wk	74.80	97.67	89.56	87.11	**98.03**	98.37	96.72	**97.81**
Lettuce_ romaine_7wk	83.60	89.35	88.69	89.36	**99.19**	99.87	94.39	**98.04**
Vinyard_ untrained	56.02	44.09	59.46	91.25	91.23	86.79	90.46	**92.68**
Vinyard_ vertical_trellis	79.84	85.50	89.11	**98.91**	97.37	91.27	91.38	98.08
OA(%)	87.14	89.87	97.51	98.87	99.61	98.85	97.16	**99.78**
AA(%)	88.36	89.60	97.10	98.09	99.04	98.46	96.89	**99.26**
ĸ × 100	84.70	87.44	95.86	97.92	98.96	98.67	97.28	**99.14**

## Data Availability

The data presented in this study are openly available at [http://lesun.weebly.com/hyperspectral-data-set.html].
